# From discovery to licensure, the Adjuvant System story

**DOI:** 10.1080/21645515.2016.1225635

**Published:** 2016-09-16

**Authors:** Nathalie Garçon, Alberta Di Pasquale

**Affiliations:** a Bioaster, Lyon, France; b GSK Vaccines, Wavre, Belgium

**Keywords:** adaptive immune response, adjuvanted vaccine, adjuvant, adjuvant system, innate immune response, vaccine development

## Abstract

Adjuvants are substances added to vaccines to improve their immunogenicity. Used for more than 80 years, aluminum, the first adjuvant in human vaccines, proved insufficient to develop vaccines that could protect against new challenging pathogens such as HIV and malaria. New adjuvants and new combinations of adjuvants (Adjuvant Systems) have opened the door to the delivery of improved and new vaccines against re-emerging and difficult pathogens. Adjuvant Systems concept started through serendipity. The access to new developments in technology, microbiology and immunology have been instrumental for the dicephering of what they do and how they do it. This knowledge opens the door to more rational vaccine design with implications for developing new and better vaccines.

## Introduction

Adjuvant Systems are combinations of immunostimulatory molecules that are designed to allow vaccines to provide better and broader protection than classical formulations containing aluminium salts. Their design was serendipitous at a time when the available knowledge did not allow to decipher their mode of action. The story of the Adjuvant Systems began with the concept of combining newly identified immunostimulatory molecules with classical adjuvants. Stable formulations were identified by mixing, matching, and using an empirical, inductive method. The value of adding Adjuvant Systems to specific recombinant antigen was demonstrated in human vaccine studies. The emerging availability of necessary tools and knowledge has allowed some aspects of their mode of action to be unravelled. This in turn permits implementation of a theoretical, deductive approach for the discovery of the next generation of adjuvants. These new developments started in the early 1990s, at a time when exponential advances in available technologies and knowledge on the interactions between the innate and adaptive immune response took place bringing new hypothesis on their mode of action, including a deeper evaluation of their positive and potentially negative effects. Adjuvant Systems are now present in 3 licensed vaccines and are part of the clinical development plan of numerous others. We describe here the journey from empirical vaccine design to the development of Adjuvant Systems, to deductive approaches that will undoubtedly bring new possibilities to improving existing vaccines and the development of new ones.

## First steps to rational vaccine design

The earliest attempts to prevent human disease through vaccination began centuries before knowledge of the existence of microbes.^1^ The early techniques of inoculation, or ‘variolation’ used to prevent smallpox were based on the empirical observation that individuals who survived infection were unaffected by subsequent exposure.^1^ These techniques were first implemented in China in the 10^th^ century using intranasal inoculation. Working in the late 18^th^ century when diseases were thought to occur as the result of a miasma, or ‘bad air’, Edward Jenner formally described that vaccination could prevent disease and thus ‘invented’ vaccination. At that time there was no knowledge of microorganisms or their role in infection, or of the immune system as the means by which our body defends itself from infections. Decades later, progress made by Louis Pasteur established that microbes cause infectious disease: a concept refined by Robert Koch, whose 4 postulates established causality between specific microorganisms and disease syndromes. This provided Louis Pasteur and later others with the basic tools to ‘invent’ vaccines.

In 1890, human cells (phagocytes) capable of ingesting and destroying pathogens were identified and attempts were made to explain the physiological interaction between human cells and pathogens. The concepts of active and passive immunity were developed, and the science of immunology was born.

The discovery of antibodies and their key role in protecting against diseases led to the production of hyper-immune serum and the use of substances called adjuvants, which were added to the antigens to maximise antibody production. The discovery of adjuvants is attributed to Gaston Ramon, a French Veterinarian and later director of the Pasteur Institute, who, while developing a diphtheria and a tetanus vaccine, established the concept that substances added to the antigen could improve the immune response and in that case, decrease the local reactogenicity.^2^ In the 1930s, aluminium became the first adjuvant to be introduced into human vaccines and continues to this day.^3^ Since then, advances in science and technology, and understanding of immune mechanisms of protection and the prevention of human disease through vaccination have been closely inter-twined. In the last 20 to 30 y the science of vaccines and immunology have brought a better understanding of host-pathogen interactions and the role played by antigens and adjuvants to elicit and modulate the desired protective immune response. Vaccine design has therefore become less empirical and more rational, with the emergence of the scientific field coined ‘vaccinology’.

### Science versus serendipity

The history of vaccine development has always been closely linked to progress in fundamental aspects of biology, chemistry, medicine, and immunology. In the same way that scientific advances stimulate the development of new hypotheses, the development of new tools to investigate those hypotheses results in the acquisition of new knowledge - this is the virtuous circle of science.

**Figure 1.**
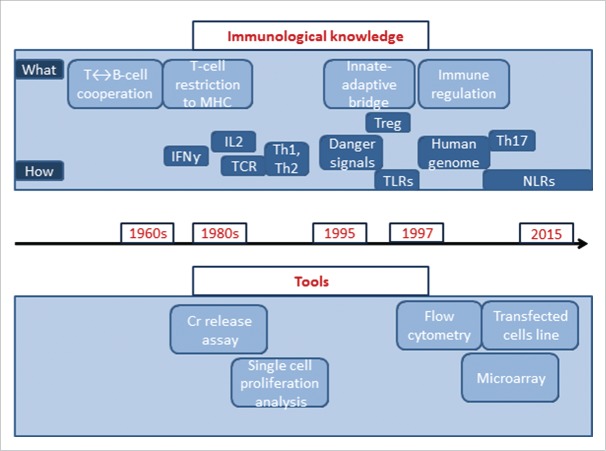
Evolution of knowledge and tools in immunology. Cr release assay = chromium release assay, MHC = major histocompatibility complex, Treg = T-regulatory cells, TLR = toll-like receptors, NLR = NOD-like receptors.

Despite the success of vaccination as a public health measure, limitations in available knowledge and technologies maintain diseases such as tuberculosis and malaria as major global public health issues. The development of vaccines against some new pathogens, such as the human immunodeficiency virus (HIV) that emerged in the 1980s, proved unsuccessful using traditional methods. Tools to improve the identification, production and purification of antigens, a deeper knowledge of the intricacies of the immune response to infection, are the major drivers of research into alternative ways of formulating effective vaccines against those and other challenging pathogens. The field of immunology, in particular, has undergone tremendous evolution over the last 25 y that has been closely followed by major advances in technology innovation to support new discoveries (Fig. 1). In parallel, the identification of new molecules with adjuvant properties stimulated intense research into the possible use of those new molecules in vaccines, and vaccines that contain novel adjuvants are emerging as a result of this research.^4^

More than 20 y ago it was recognized that novel adjuvants would be key to the development of new vaccines against difficult pathogens of global public health importance, because the classical aluminum approach was unable to induce the desired immune response. Here we relate the journey from the first empirical research of adjuvants to the concept of GSK's Adjuvant Systems (defined as AS) and the demonstration of their clinical value.

## The beginning of the journey

By the mid-1980s the quest to develop a vaccine against HIV infection had begun. Classical vaccines proved inadequate in the face of efficient evasion of host immune responses by HIV, the rapidity of viral replication, and a high rate of mutations and recombinations leading to escape mutants. Numerous approaches were tested without success; these included combinations of recombinant antigens with the addition of an adjuvant (aluminum, QS-21 [Quillaja saponaria Molina: fraction 21 (Antigenics Inc., a wholly owned subsidiary of Agenus Inc., Lexington, MA, USA)], MF59, liposomes, or MPL [3-deacylated monophosphoryl lipid]), DNA plasmid vaccines, and viral vector vaccines using pox viruses, adenoviruses and others.^5,6^

**Figure 2.**
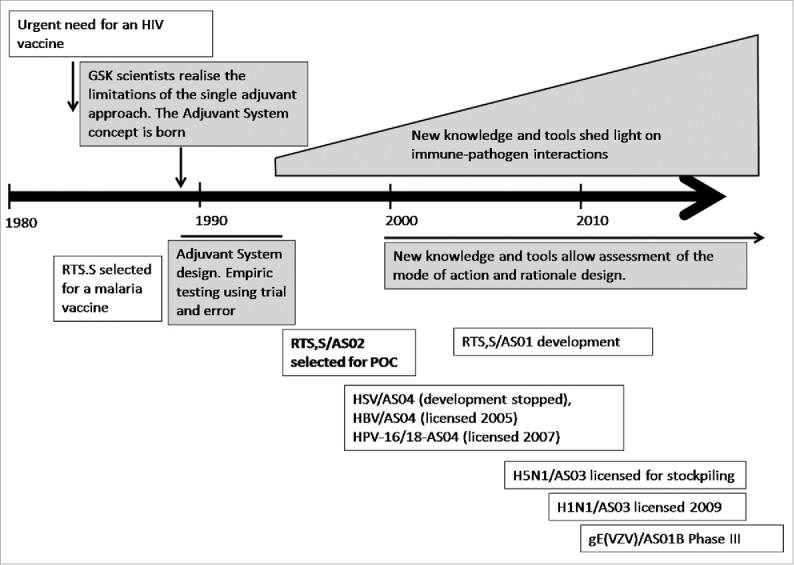
Adjuvant Systems: key developmental milestones. POC = proof of concept.

It was at the same time that the first human recombinant vaccine (containing recombinant hepatitis B surface antigen) was licensed, and that a recombinant malaria antigen candidate was developed. Although considerable expertise in recombinant technology was acquired, it had become clear that aluminum adjuvants were not sufficient to induce and modulate specific arms of the immune response (humoral and cellular). New adjuvants with specific immune effects were becoming increasingly critical for successful vaccines against challenging pathogens such as HIV. By 1990, it was realized that the use of single adjuvants was not sufficient for an HIV vaccine to be successful, but there was a continued belief that the adjuvant approach was of value. This led to a new paradigm that of the potential power of combinations of immunostimulants. Thus began the identification of molecules that, if proven compatible, could be combined into Adjuvant Systems with the goal of generating protective immune responses (Fig. 2).

## The adjuvant system concept

The Adjuvant System approach was seen as an avenue to design vaccines against difficult pathogens such as HIV and malaria. In the absence of precise knowledge on their mode of action, molecules were selected on the basis of previous observations generated with different antigens. Among a range of adjuvant molecules, those that were best characterized and most promising because of their immuno-enhancing properties were selected. Adjuvants such as MPL and its various formulations discovered in the early 1980s, QS-21 identified in the late 1980s, and CpG identified in 1995, had already been tested in humans. As they were known to promote different aspects of the adaptive immune response, these molecules were named ‘immune-modulating molecules’.^7^

By combining the various molecules (MPL, QS-21, CpG) with classical adjuvants (aluminum, liposomes, oil-in-water emulsions) to form the Adjuvant Systems, it was expected to obtain, at the very least, an additive effect on the adaptive immune response. However, it was hoped that a complementary or even synergistic effect on the resulting specific responses (cellular as well as humoral components) would occur, and potentially an impact on the breadth of the immune response would be observed.

With the use of the Adjuvant System, the adjuvanted vaccine had to induce a stronger effective immune response against the antigen as compared to no adjuvant or aluminum-based adjuvant formulations, demonstrate an acceptable reactogenicity profile in the target population, and be physically compatible with the antigen. Ease and reproducibility of the vaccine formulation at the manufacturing level were also key elements considered when selecting an Adjuvant System–antigen combination. It was quickly realized that the molecular environment (i.e., the vaccine formulation) was as important as the molecule itself in the creation of an efficient and sustainable Adjuvant System.

Over a 5-year period 10 different Adjuvant System families were designed based on mixing and matching immune-stimulatory molecules and classical adjuvants, followed by the identification of stable formulations. Currently, 5 of these adjuvant families have been investigated in clinical trials, and 3 are in vaccines that are licensed, or close to licensure (Table 1). AS01, AS02, AS04 and AS15 all contain MPL, a potent agonist of toll-like receptor (TLR) 4. AS04 induces a TH1-biased immune response and is thus evaluated in vaccines targeting viral infections. AS02 contains MPL combined with QS-21 in an oil-in-water emulsion, and induces strong humoral and T-cell-mediated responses. AS02 has been evaluated in vaccines targeting complex pathogens requiring a strong T-cell response. AS01 contains MPL, QS-21 and liposomes, and was designed to strengthen the CD8+ response still further. AS01 is currently used in a candidate malaria vaccine and a candidate zoster vaccine. AS03 contains alpha-tocopherol (Vitamin E) and squalene, and induces a marked antibody response, and is used in vaccines were antibody-mediated protection is important. AS15 contains several immunostimulants (MPL, QS-21 and CpG) with the aim of inducing anti-tumor activity in cancer immunotherapeutics.^8^ Examples of how some of these Adjuvant Systems were designed and the particular challenges that were overcome are given below.

**Table 1.** GSK's Adjuvant Systems.

Adjuvant System	Composition	Vaccines licensed or in Phase III trials	Vaccines in Phase I or II trials	Development discontinued
AS01	A combination of immunostimulants QS-21 and MPL with liposomes	Malaria vaccine Herpes zoster vaccine	Malaria next generation COPD exacerbations associated with non-typeable Haemophilus influenzae and Moraxella catarrhalis Tuberculosis vaccine HIV vaccine	—
AS02	A combination of immunostimulants QS-21 and MPL with an oil in water emulsion	—		HIV vaccine Tuberculosis vaccine Therapeutic melanoma vaccine Malaria vaccine.
AS03	A combination of an oil in water emulsion with alpha-tocopherol (Vitamin E) as immuno-enhancing component	Pre-pandemic H5N1 vaccine Pandemic H1N1 influenza vaccines (*Arepanrix™, Pandemrix™*)	—	
AS04	MPL is adsorbed onto aluminum hydroxide or aluminum phosphate, depending on the vaccine with which it is used	Human papillomavirus vaccine (*Cervarix™*) Hepatitis B for pre- and haemodialysis patients (*Fendrix™*)	—	Herpes simplex vaccine
AS15	A combination of immunostimulants CpG 7909, QS-21 and MPL with liposomes	—	—	MAGE-A3 Cancer Immunotherapeutics: melanoma and non-small-cell lung cancer vaccines

QS-21: Quillaja saponaria Molina: fraction 21. (Antigenics Inc., a wholly owned subsidiary of Agenus Inc., Lexington, MA, USA).

MPL: 3-deacylated monophosphoryl lipid.

CpG7909: an immunostimulatory nucleotide.

COPD: Chronic Obstructive Pulmonary Disease

### AS03: The challenge of sterile filtration

Oil-in-water emulsions have historically been considered to be more efficient at inducing high antibody titres than aluminum, and were investigated for use in Adjuvant Systems. For each oil-in-water formulation specific physico-chemical criteria had to be met to ensure manufacturability and ease of production. Adjuvants based on an oil-in-water emulsion had to be amenable to 0.2 micron sterile filtration to ensure sterility of the final product. This meant that the emulsion droplets had to be smaller than 200nm, the pore size of the filtration membrane – and had a narrow size range distribution. Furthermore, it was foreseen early on that components such as alpha-tocopherol (Vitamin E) would need to be introduced to the emulsion, based on its known immune-enhancing properties.^9^

More than 60 different oil-in-water emulsions were assessed for their suitability for use in vaccines (Table 2). These emulsions were based on a limited number of defined ingredients selected for their ability to produce an emulsion, as well as their immune-enhancing properties. As the emulsion needed to be sterile filterable and chemically and physically stable over time, classical pharmaceutical tests were applied to the candidate emulsions. The selected emulsions had a droplet size amenable to sterile filtration (less than 200 nm), and showed stability over time (no destabilization as defined by separation of phases after several cycles of freezing and thawing). One emulsion was selected and forms the basis of AS03 (Table 1).

**Table 2.** Impact of composition on the physical properties of oil-in-water emulsions (a subset of more than 60 tested formulations). Key requirements were stability over time after freeze/thaw, and sterile filterability (<200 nm). Shaded area represents the selected emulsion known as AS03.

	Vehicles 2-fold concentrated					
Emulsion	Tocopherol %	Squalene %	Tween 80%	Span 85%	Lecithin %	Size (nm)
26	5	5	0.4	0	0	500 (90–100%) 800 (0–10%)
26.1	5	5	0.4	0	0.1	500
63	5	5	0.6	0	0	500
64	5	5	0.8	0	0	500
61	5	5	1	0	0	250–300
62	5	5	2	0	0	180
40	5	5	0.4	1	0	500 (80–100%) 800 (0–20%)
40.1	5	5	0.4	1	0.1	500
60	5	5	1	1	0	300
65	5	5	0.4	1.5	0	500
66	5	5	0.4	2	0	500

### AS04: The challenge of MPL insolubility in water

To overcome the problem of its water-insolubility, MPL was adsorbed on aluminum. The dogma at the time was that both antigen and immunostimulant needed to be physically associated to achieve an optimum immune response. Therefore, antigen was added to adsorbed MPL in a second sequential adsorption step, but this could result in potential variation in the MPL: antigen ratio on individual particles, which could have made characterization of the final product difficult. Evaluation of the formulation in animal models showed that an equivalent immune response was obtained whether the antigen and MPL were sequentially adsorbed on the same particles, or adsorbed separately on different particles and then combined. Thus, to ensure reproducibility of the formulation and minimize variation in the MPL and antigen amount per alum particle, it was decided to adsorb MPL and the antigen(s) separately, and then mix the pre-adsorbed bulk to reach the final formulation

### AS01: The challenge QS-21 lytic activity

**Figure 3.**
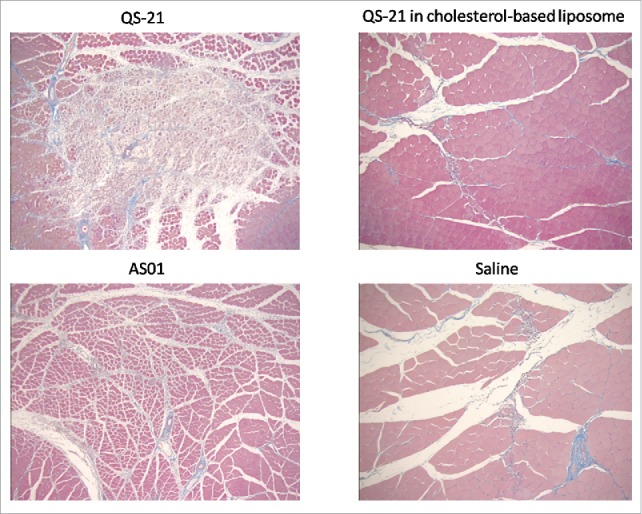
Lytic action of QS-21 is abrogated in cholesterol-based liposomes.

QS-21 is a lytic saponin fraction of QuilA and needed to be formulated in such a way as to abrogate its intrinsic lytic activity (Fig. 3). This was achieved by using cholesterol-based liposomes as a delivery platform. Interestingly, the widely held belief at the time was that the immune-stimulant effect of QS-21 was due to its ability to form pores in the cell membrane, hence its lytic activity. It was thought that formulating QS-21 in a way that removed this activity would eliminate its adjuvant effect. On the contrary, cholesterol-quenched QS-21 was shown to be as immunostimulating as free QS-21, calling into question the original dogma.

Quenched QS-21 also proved to have higher stability than the original formulations. The stability of quenched QS-21 was shown to be significantly increased at a pH above 6.4 while native QS-21 is susceptible to hydrolysis at a pH above 6.4 and loses its adjuvant effect. Formulations containing quenched QS-21 were demonstrated to be stable for several hours at pH above 8.8 and at temperatures above 37°C. These formulations became the AS01 Adjuvant System family (Table 1).

As typical in innovative processes, it is the technical hurdles encountered along the way and the ability to find sustainable solutions that generate technology improvements. Overcoming those hurdles also challenges, and either confirms or disproves widely held beliefs, advancing knowledge in the field. While the development of adjuvants was initially stimulated by the need for an HIV vaccine, the absence of a validated target HIV antigen, as well as an appropriate challenge model meant that the proof of concept of the first Adjuvant Systems was evaluated in another model: the malaria vaccine candidate.

## Proof of concept: The malaria vaccine

Work on a malaria vaccine started more than 30 y ago. The development of a malaria vaccine is particularly complex due to the parasite cycle, since it resides within several human tissues and presents different patterns of antigen expression during its life-cycle.^10^ By themselves, antibodies are not sufficient to clear malaria from the host.^11^ A co-ordinated immune response of sufficient magnitude and targeted against a relevant antigen is required, with humoral and cellular components that are activated at specific stages of the parasite life-cycle, as well as at specific anatomic locations to induce protection.

In the early-to-mid-80s the recombinant RTS,S antigen (a hybrid antigen in which a portion of the circumsporozoite protein is fused to hepatitis B surface antigen), targeting the pre-erythrocytic stage of the malaria parasitic life-cycle was selected for further vaccine development.^12^ By itself RTS,S is only weakly immunogenic. The addition of aluminum salts was ruled out early in development, as this adjuvant did not induce a protective immune response when incorporated with the RTS,S antigen candidate.^13^ Alternative adjuvants were required, but knowledge at the time was not sufficient to predict which adjuvants would induce an optimal response. Pre-clinical trials were conducted with several candidate Adjuvant Systems, with successful candidates tested further in human challenge trials. In contrast to HIV, it was the availability of a suitable, albeit weakly immunogenic antigen candidate (RTS,S), a suitable human challenge model, and the clear need for an effective malaria vaccine that led to the selection of malaria for the proof-of-concept of the Adjuvant System approach.

The ultimate development goal was to produce a malaria vaccine that induced a multi-tiered immune response: one that induced neutralising antibodies capable of clearing sporozoites when present in the blood (or at least decreasing the parasite load), and preventing hepatocyte invasion; and a strong cell-mediated immune (CMI) response capable of interfering with the intrahepatic stage of the parasite by killing infected hepatocytes, or impairing the development of intrahepatic parasites.

A confirmation of the critical importance of adequate formulation and of the value of an Adjuvant System referred to as ‘AS02’ (an oil-in-water emulsion with MPL and QS-21) for the malaria vaccine was obtained in a challenge trial conducted in collaboration with the Walter Reed Army Institute of Research.^14^ Compared to vaccine adjuvanted with AS03 (an oil-in-water emulsion) or AS04 (a combination of aluminum and MPL), the malaria vaccine adjuvanted with AS02 induced protection in 6 out of 7 volunteers, and was thus selected for further clinical studies.^15^ Assessment of the immune response showed that AS02 elicited high antibody responses with levels that were similar to AS03 and higher than AS04, with the highest number of subjects showing a CMI response as compared to the 2 other formulations.^16^ Subsequent clinical trials conducted with the RTS,S/AS02 formulation showed evidence of partial protection against malaria infection and severe disease in adult populations,^17^ children^18^ and infants.^19^

During RTS,S/AS02 development, work was also done on AS01, a liposome-based Adjuvant System using QS-21 and MPL, and with which the induction of a T-cell response was improved.^20,21^ Research in mice and rhesus monkeys showed that AS01 induced significantly superior antigen-specific cytotoxic T-lymphocyte and interferon-gamma (IFN-γ)-producing CD4+ T-cell responses than AS02, while humoral responses generated by both formulations remained equivalent.^22^ In rhesus monkeys, AS01 induced a CMI response characterized by a Th1-biased profile and a higher proportion of IFN-γ-secreting lymphocytes compared to AS02.^22^ Finally, a trend toward better protection with an AS01-adjuvanted RTS,S vaccine compared to the AS02 formulation was observed in human challenge studies.^23,24^ Based on these results and further confirmation in clinical settings,^25-27^ development of RTS,S/AS02 was stopped and the RTS,S/AS01-adjuvanted vaccine was selected for testing in Phase III studies.^28^

## An adjuvanted Herpes simplex vaccine

Parallel to the work being undertaken in the development of a malaria vaccine, a vaccine against Herpes simplex virus (HSV) type 2 was being developed. The HSV vaccine was made possible by the availability of a viable candidate antigen (the truncated version of glycoprotein D surface antigen, gD2), and the existence of a guinea pig challenge animal model. Prevention of HSV is challenging; protection needs to occur in mucosal tissue, and the virus has a latent phase during which it resides for long periods of time in dorsal root ganglia until re-activation.

It was found that the HSV recombinant protein gD needed to be adsorbed on aluminum in order to achieve an immune response of good quality and consistency. In preliminary experiments conducted in guinea pigs, the aluminum-adjuvanted formulation led to an improved response against clinical symptoms of HSV, but did not provide full protection.^29^ Because of its known properties of enhancing the magnitude of the antibody response, MPL was added to the formulation. This allowed development of the final form of the aluminum-MPL Adjuvant System known as AS04.

Studies conducted in guinea pigs established the proof-of-concept, and the first real evidence of the possibility to protect at a mucosal level after parenteral vaccination. Immunization with HSV/AS04 provided good protection again primary HSV disease and was associated with reduced viral shedding in the genital tract as compared to the controls, but did not prevent mucosal infection.^30,31^ Compared to an aluminum-adjuvanted vaccine without MPL, HSV/AS04 prevented more recurrent HSV disease.^30^

Two initial clinical trials conducted in the 1990s that enrolled more than 1500 men and women showed that HSV/AS04 conveyed highly significant protection (73% in one study and 74% in the other study) in a specific population: women (but not men) who were seronegative for HSV-2 and HSV-1.^32^ However, a larger Phase III clinical trial in more than 8000 HSV-1 and HSV-2 negative women failed to demonstrate efficacy for reasons that remain unclear.^33^ Later investigations indicated that the vaccine dose and formulation were unlikely to have contributed to the Phase III results.^34^ The only factor identified as different between the initial studies and the later Phase III trial was that the first studies enrolled discordant couples: the enrolled HSV-seronegative women had a partner with HSV, whereas the later study enrolled all seronegative women regardless of the HSV status of their partner.^33^ Differences in HSV exposure, and potentially a difference in underlying resistance to infection between these 2 populations of women may have contributed to the results observed in Phase III.^33^

On the basis of the Phase III efficacy results further development of the HSV/AS04 vaccine was interrupted. The unsuccessful attempt to develop an HSV vaccine using the AS04 Adjuvant System illustrates how apparently subtle differences in susceptibility and exposure can impact on the success or failure of a vaccine. The HSV/AS04 story also serves to highlight the gaps that still exist in our knowledge about the inner workings of the immune system.

## Demonstration of the value of AS04

### Hepatitis B virus vaccine

Knowledge of the immune-stimulating capabilities of AS04 was extended to other existing vaccines for which a stronger antibody response was required: notably the hepatitis B virus (HBV) vaccine. Recombinant HBV vaccines adjuvanted with aluminum salts show good efficacy in preventing HBV infection.^35^ Nevertheless, in some populations the immune response to vaccination is impaired. Patients with end-stage renal disease have an increased risk of acute HBV infection, an increased risk of progression to chronic hepatitis, and are relatively poor responders to aluminum-adjuvanted HBV vaccines.^36^ These individuals require multiple vaccinations with higher doses than required for healthy individuals in order to achieve and maintain seroprotection. An AS04-adjuvanted recombinant HBV vaccine was developed with the aim of improving the magnitude, kinetics and quality of the antibody response in patients receiving haemodialysis. Studies in mice showed that HBV/AS04 induced an increase in antibody titres compared to the classical formulation which contained aluminum alone.^37^ In clinical trials, HBV/AS04 induced a higher, and more durable antibody response, with enhanced CMI responses in pre-haemodialysis and haemodialysis patients compared with multiple double doses of the classical formulation.^38^ Moreover, the administration of one booster dose of HBV/AS04-adjuvanted vaccine at 42 months increased antibody concentrations by 199-fold, which was significantly higher than post-booster response (increase of 64-fold) achieved by the classical vaccine.^39^

It is noteworthy that the availability of a well-accepted serological correlate of protection against HBV infection substantially accelerated the development of the HBV/AS04 vaccine, which was licensed in 2005 under the trade name *Fendrix*™, and was the first licensed AS04-adjuvanted vaccine in Europe. This is in contrast to prevention of human papillomavirus (HPV) infection for which no serological correlate of protection existed; although subsequently it is now generally accepted that neutralising antibodies are the main determinant of protection. Thus, while development of an AS04-adjuvanted HPV vaccine proceeded in parallel with the HBV/AS04 development, the HPV-16/18-AS04-adjuvanted vaccine (*Cervarix*™) was licensed 2 y later, in 2007.

### Human papillomavirus vaccine

HPV persistent infection is a prerequisite for the development of cervical cancer.^40^ Among the 15 HPV oncogenic strains identified, HPV-16 and HPV-18 account for overall 70% of cervical cancers, and the closely related strains HPV-45 and -31 for another 10%.^41^ Many HPV infections are asymptomatic and are cleared by natural immunity, but persistent infection is a precursor to the development of cervical precancerous lesions and cervical cancer. HPV is typically undetected by the adaptive immune system as the virus invades squamous cells of the skin and mucosa, where no viral cytolytic activity occurs.^42^ The vaccine developed was to provide strong and long-lasting protection against incident and persistent infections with the HPV-16 and HPV-18 strains associated precancerous lesions. This was thought to be possible if high levels of neutralizing antibodies could be induced, which would then transudate from the blood to the cervical mucosa.^43^ AS04 was selected as the adjuvant of choice because it contains aluminum and MPL, which was known to induce higher antibody levels than aluminum alone, and has an acceptable safety profile.

This selection was confirmed in initial experiments in mice and monkeys which showed significantly higher titres of HPV-specific antibodies than a formulation which only contained aluminum salt as adjuvant.^44^ In order to evaluate the quality of the humoral response, specific HPV neutralizing antibodies were analyzed in monkeys. Again, the formulation with AS04 induced higher titres of neutralizing antibodies compared to the aluminum salt formulation.^44^

In clinical trials, the efficacy of HPV-16/18-AS04-adjuvanted vaccine was conclusively demonstrated and long term efficacy (up to 8.4 y to date) has been shown for women vaccinated between 15 and 25 y of age.^45-50^ A degree of cross-protection has been demonstrated against strains phylogenetically related to 16 and 18, such as 31 and 45: vaccine efficacy against persistence infection at 6 months was 77.5% (95% confidence interval [CI] 68.3–84.4) for HPV-31 and 76.1% (59.1–86.7) for HPV-45.^45^ At 48 months after vaccination, vaccine efficacy against cervical intraepithelial neoplasia lesions of level 3 or above was 93.2% among women who were initially HPV-naïve (Total vaccinated cohort analysis).^51^ First real-world data from universal mass vaccination with HPV-16/18-AS04-adjuvanted vaccine in Scotland confirmed the significant decrease in HPV-16 and 18 prevalence among cervical samples from vaccinated women and cross-protection against non-vaccine types including HPV-31, −33, and −45.^52^ These results provide early evidence that vaccination with HPV-16/18-AS04-adjuvanted vaccine could offer benefits in reducing cervical pre-cancer and cancer that are over and above those expected from preventing lesions caused by HPV type-16 and 18 alone.

Comparison of the immunological response induced by the AS04-adjuvanted HPV16/18 vaccine with another licensed aluminum salt-adjuvanted HPV6/11/16/18 vaccine in women aged 18–45 showed that HPV-16/18-AS04 induced higher levels of neutralizing HPV-16 and HPV-18 antibodies, and higher frequencies of memory B cells.^53^ A higher percentage of HPV-16/18-AS04 recipients had a CD4+ T-cell response, and HPV-16/18-AS04 recipients had higher mean frequency of circulating antigen specific CD4+ T-cell than HPV6/11/16/18 vaccine recipients.^53^ Both neutralizing antibodies and stronger T-cell responses may explain the cross-protective features of HPV-16/18-AS04. Since it is believed that antibody levels at the cervix are most relevant for protection against HPV infection, it is plausible that higher serum antibodies may be associated with transudation of more antibodies through the cervix epithelium.^54^ However, the clinical significance of differences in serum antibody levels achieved using HPV-16/18-AS04-adjuvanted vaccine and aluminum salt-adjuvanted HPV6/11/16/18 vaccine remains to be determined, since both vaccines have shown high efficacy in preventing HPV infection and pre-cancerous lesions.^55^ The induction of a robust B-cell memory response by HPV-16/18-AS04-adjuvanted vaccine is anticipated to support the persistence of the antibody response and hence, long-term protection.^44^

## Demonstration of the value of AS03

### The pandemic experience

Around 10 y ago the World Health Organization (WHO) identified the avian H5N1 influenza strain as the most probable strain for a future influenza pandemic.^56^ In the event of an influenza pandemic, it is conceivable that global vaccination will be required, with rapid escalation of vaccine production and distribution to unprecedented levels. A pandemic influenza vaccine thus needs to fulfil 4 key criteria in order to be effective at a global level^57^: 1) the vaccine must induce robust immunogenicity using a low dose of antigen across all age-groups; 2) the immune response induced by vaccination should be broad and durable; 3) the safety and reactogenicity profile of the vaccine should be acceptable in the pandemic context; 4) it should be able to be produced rapidly and in large quantities to support worldwide supply.

By using an Adjuvant System, it was considered feasible to develop an H5N1 pandemic vaccine of sufficient immunogenicity to allow antigen-sparing, while retaining immunogenicity and cross-reactivity in all age groups. During the search for an appropriate Adjuvant System for use in a malaria vaccine, a clinical study demonstrated that the antibody response to RTS,S/AS03 was as high as that achieved using AS02, even though clinical protection against malaria was not as high.^15^ AS03 was an attractive candidate for a pandemic influenza vaccine due to its ability to induce high antibody titres, its compatibility with the influenza vaccine antigen, and importantly, the ability to manufacture it quickly and at large scale in the event of an urgent need for large quantities of vaccine.

An heterologous challenge study in ferrets demonstrated a strong and cross-reactive immune response elicited by an AS03-adjuvanted H5N1 vaccine.^58^ An important observation made during H5N1/AS03 vaccine development was that AS03 increased the diversity and magnitude of the antibody response, as shown with the H5N1/AS03-adjuvanted vaccine (Vietnam strain) (which protected against the cross-clade Indonesia strain). All animals receiving 2 doses of the adjuvanted vaccines (containing only one-quarter of the antigen amount usually administered in the context of seasonal influenza vaccination) survived a lethal heterologous challenge, whereas all animals in the control group died.^57^ A subsequent study demonstrated that humoral responses elicited after a second H5N1/AS03 dose were long-lasting.^59^

The formulation of an H5N1 candidate pandemic strain with AS03 demonstrated the ability of this Adjuvant System to overcome key clinical challenges in pandemic vaccine development. In clinical trials, healthy adults (aged 15–60) showed a strong antibody response to two H5N1/AS03 doses containing as little as one-quarter of the antigen dose typically contained in seasonal influenza vaccines.^60^ Immunogenicity of H5N1/AS03 was also demonstrated in children and infants,^61^ and cross-clade immunity with the AS03-adjuvanted formulation was demonstrated.^62,63^ The H5N1/AS03 vaccine was approved for use in case of a H5N1 pandemic in individuals aged 18 y and above, by the US Food and Drug Administration in 2013.^64^

Contrary to predictions however, the next pandemic influenza strain was not an H5N1 strain but an H1N1 strain of swine origin. Due to the appearance and rapid spread of this new strain, WHO declared a level 6 pandemic alert on June 11, 2009.^65^ The large experience already gained during the development of H5N1/AS03 guided the development of an H1N1/AS03 pandemic influenza vaccine. This was achieved rapidly and the H1N1/AS03 vaccine was licensed in Europe and internationally for prophylaxis against the 2009/2010 swine influenza pandemic since 2009.

The H1N1(2009)/AS03-adjuvanted vaccine met regulatory acceptance criteria for vaccine-homologous antibody responses in clinical trials. Studies conducted in adults, children and infants showed that H1N1/AS03 was associated with increased immunogenicity and an increased seroconversion rate as compared to non-adjuvanted whole virion vaccine.^62,66-71^ Studies undertaken in several European countries, Canada and Australia, have shown vaccine effectiveness estimates between 62% and 100% for laboratory-confirmed influenza (depending on the age group studied and the presence of risk factors for severe influenza); and 90% against influenza hospitalisation.^72-80^ The value of AS03 was demonstrated in the context of the H1N1 pandemic, with all criteria for a successful pandemic vaccine achieved.

## Demonstration of the value of AS01

### Herpes zoster vaccine

Varicella zoster occurs when T-cell memory responses become dysfunctional; most commonly due to immune senescence associated with aging or immune suppression.^81^ Reduced T-cell numbers and suboptimal T-cell activity allow reactivation of latent herpes zoster virus which leads to clinical disease. Prevention of reactivation can be achieved by vaccines that increase specific CMI responses.^82^

The value of the AS01 and AS02 Adjuvant System families in inducing multi-tiered adaptive immune responses that include a strong CMI component was demonstrated during the development of the candidate malaria vaccine.^21^ The potential of AS01 and AS02-adjuvanted vaccines vs. an aluminum-adjuvanted vaccine (all containing the varicella zoster virus glycoprotein E [gE] antigen), to prevent zoster, was first tested in a mouse model.^83^ The gE/AS01_B_ formulation induced markedly higher CMI responses compared to aluminum-adjuvanted gE and was selected for investigation in human studies. This is the first indication of the value of the Adjuvant System approach in the context of a vaccine targeted for use in an immune senescent population that has already encountered the virus, which is present in a latent form.

The gE/AS01_B_ formulation was tested in a Phase I/II study in adults and showed antigen-specific humoral and T-cell responses that were significantly higher compared to unadjuvanted varicella vaccine.^84^ In a large, placebo-controlled Phase III study, the gE/AS01_B_ has recently been shown to have an acceptable safety profile, with efficacy against herpes zoster in adults ≥50 y of age of 97.2% (95%CI 93.7–99.0),^85^ and is now undergoing submission to competent authorities.

## Adjuvant systems: Safety aspects

The evaluation of vaccine safety is as an integral part of the vaccine development program as is the assessment of immunogenicity and efficacy. Vaccines typically contain an active ingredient/s (antigen) and other molecules such as preservatives, stabilisers (lyophilized vaccines) and adjuvants, and could also contain residual traces of substances used in the process of manufacturing. As for all vaccines, the assessment of the safety profile of adjuvanted vaccines is made on the final product administered to humans, not on the individual components.

Vaccine safety is assessed pre-clinically, at all phases of clinical development, and continues after licensure throughout the life of the product. Regulatory authorities require thorough preclinical testing of vaccines, including those formulated with new adjuvants, in order to identify potential adverse effects. Safety assessment during clinical trials may be guided by pre-clinical study results, the characteristics of the target population, and experience with similar vaccines or other vaccines containing the same adjuvant. Because clinical trials usually enrol limited numbers of subjects, rare serious adverse events may not be identified prior to licensure. It is for this reason that post-approval surveillance mechanisms are in place for the continuous assessment and monitoring of vaccine safety in the broader population.

**Figure 4.**
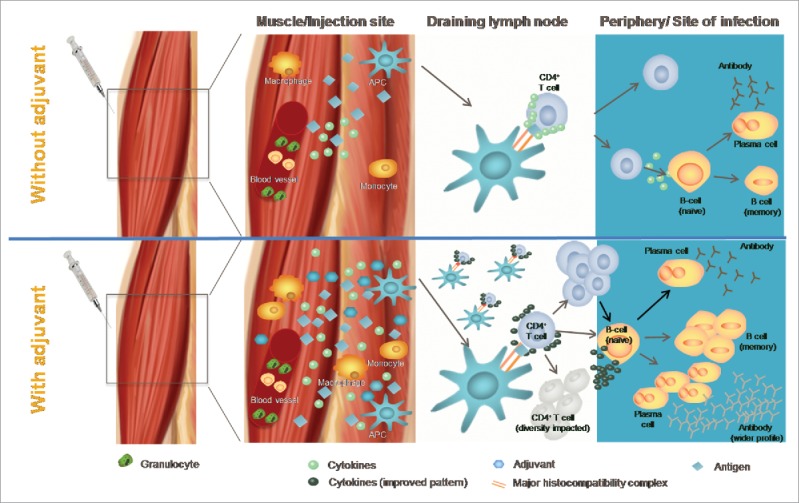
General mode of action of an adjuvant (adapted from^104^).

Through their impacts on the innate immune response, adjuvants can stimulate the induction of cellular and humoral adaptive immune responses (Fig. 4). Thus, there is a theoretical risk of potentially developing a vaccine induced immune-mediated disease.^86^

New knowledge acquired as experience with the vaccine product grows after licensure stimulates further research to explore aspects of vaccine safety at the molecular and cellular level, as well as in targeted studies or epidemiological investigations. Pre-clinical studies in appropriate animal species may help to evaluate adverse events requiring particular attention during clinical development or post-approval. In this context, understanding the mode of action of a specific adjuvant may provide additional data beyond that available from observations made during pre-clinical and clinical studies. The data from these investigations is used to continuously assess the benefit-risk profile of the vaccine during its life-cycle, and can also influence the development of new vaccines.

### Current experience with approved vaccines adjuvanted with AS04

AS04 is present in GSK's HPV vaccine and HBV vaccine for patients with renal insufficiency. The AS04-adjuvanted HBV vaccine designed for patients with renal insufficiency demonstrated a safety profile similar to standard aluminum adjuvanted HBV.^87^

Safety analyses of the AS04-adjuvanted HPV vaccine demonstrated that this vaccine is generally well tolerated across all evaluated age groups. Solicited local symptoms such as pain, redness or swelling are more common in recipients of HPV-16/18-AS04-adjuvanted vaccine than in recipients that received the aluminum-adjuvanted HPV vaccine. This observation is in line with the local and transient direct activities of AS04.^88^ Rates of solicited general symptoms tended to be slightly higher in the AS04-adjuvanted HPV vaccine group compared to control group.^89^

In a pooled analysis of clinical trial data from more than 57,000 girls and women, the nature and incidence of adverse events reported up to 30 d after vaccination, medically significant conditions, serious adverse events and potential-immune-mediated diseases were similar among HPV-16/18-AS04-adjuvanted vaccinees and controls who received another vaccine.^90^ Post-approval licensure surveillance activities including enhanced safety surveillance in countries where HPV-16/18-AS04-adjuvanted vaccine is used, analysis of adverse events of interest such as the occurrence of potential-immune-mediated diseases, review of pregnancy outcomes in instances when vaccination occurred inadvertently around early pregnancy, and observed-versus-expected analyses to evaluated the occurrence of specific adverse events, confirm the acceptable benefit-risk profile in adolescent girls and women.^91^

### Licensed vaccines adjuvanted with AS03

Since the H1N1 influenza pandemic in 2009, more than 90 million doses of AS03-adjuvanted H1N1 vaccine have been administered worldwide in more than 47 countries, with about 4.7 million doses administered to children. Thorough safety surveillance carried out by vaccine manufacturers and national public health agencies during the 2009/2010 pandemic season showed a positive benefit-risk profile for the vaccine.

In early 2010 GSK became aware of reported cases of narcolepsy following vaccination with the AS03-adjuvanted H1N1 pandemic vaccine, *Pandemrix*™. A body of data including spontaneous reports and results of epidemiological studies conducted in several countries in Europe suggested an increased risk of narcolepsy in vaccinated individuals vs. the unvaccinated population.^92^ However, further research is ongoing to better understand the potential contribution of AS03-adjuvanted H1N1 vaccines to the development of narcolepsy and how other factors (genetic, environmental, circulating infections) associated with narcolepsy may have played a role. Some recent publications suggest this may be related to aspects of the vaccine viral protein,^93,94^ although clear evidence supporting causality is lacking so far. The vaccine has not been in use since the 2009/2010 pandemic and its license has recently expired.

A meta-analysis of trials conducted with AS01, AS02, AS03 and MF59 in more than 25,000 children showed higher reactogenicity of the adjuvanted vaccines compared with control vaccines (adjuvanted or unadjuvanted), but no consistent pattern was observed.^95^ No safety concerns were identified and no increased risk of adverse event of special interest; febrile convulsion, immune-mediated disease, or new onset of chronic disease was found.

## The emergence of new knowledge

Studies in biology require scientists to develop new technologies to assess and study new hypothesis. Those tools allow the generation of data that extend beyond the original question, bringing new hypotheses that require new tools to be able to answer them. This is the self-perpetuating cycle of scientific research.

It was during the 1990s that the pivotal role of the innate immune response in the generation of adaptive immunity was discovered (Fig. 1).^96^ Identification of the role played by dendritic cells in the induction of a persistent immune response, as well as recognition of the role of specific molecules present on all pathogens called ‘Pathogen-associated molecular patterns', opened new areas of vaccine adjuvant research.^97,98^ The importance of these findings was recognized when the 2011 Nobel Prize for Immunology was shared between BA Beutler and JA Hoffman who had conducted pivotal work on activation of the innate immune responses, and with RM Steinman who had discovered the dendritic cell.^99^ For vaccine scientists, this knowledge marked the transition between inductive development, to the deductive rational design of Adjuvant Systems using existing molecules such as MPL, CpG, and Flagellin, and new molecules such as Sting, NOD-like receptors and other ligands to established tailored-made adjuvants systems. This also opened the door to a better understanding of the mode of action of all vaccines, from live-attenuated vaccines that contain intrinsic immune defense triggers, the so called danger signals (part of the vaccine antigens) to recombinant adjuvanted vaccines, containing exogenous (added to the vaccine) immune defense triggers. The immune system recognizes those molecules via their interaction with a range of receptors including toll-like receptors and NOD-like receptors, resulting in a specific downstream adaptive response.^100^ It became clear that the interactions of immune-stimulatory molecules with specific receptors expressed by antigen presenting cells (such as toll-like receptors) was one of the key elements bridging the innate and adaptive immune responses, influencing the profile of the response induced.^101^

New knowledge about the interactions between the innate and adaptive immune response allowed scientists to better understand Adjuvant Systems, including a deeper evaluation of their positive and potentially negative effects. A better understanding of the mechanism of action of Adjuvant Systems allowed a more in-depth assessment of the safety profile and the potential impact of the formulation on reactogenicity and safety. In addition, the advantages and disadvantages of animal models in evaluating Adjuvant Systems also became better understood in light of qualitative and quantitative differences in innate receptors on immune cells from animals and humans.

A concrete example of this process is the HPV/AS04 vaccine. During discussions with licensing authorities it became clear that additional data describing its mode of action would be needed to support the approval process of the new Adjuvant System containing vaccine. This prompted additional safety analyses to be performed and research to investigate the mode of action of AS04 *in vitro* and *in vivo*. The adjuvant activity of AS04 was found to be strictly dependent on AS04 and the HPV antigens being injected at the same intramuscular site, together or within 24 hours of each other.^88^ During this period, AS04 induced transient local NF-kappaB activity and cytokine production.^88^ This led to an increased number of activated Antigen-loaded dendritic cells and monocytes in the draining lymph node, which further increased the activation of antigen-specific T-cells. AS04 was also found to directly stimulate those antigen-presenting cells *in vitro* but not directly stimulate CD4+ T-or B- lymphocytes. These AS04-induced innate responses were primarily due to MPL. Aluminum salt appeared to prolong the cytokine responses to MPL at the injection site. Together these results support a model in which the addition of MPL to aluminum salt enhances the vaccine response by rapidly triggering a local cytokine response leading to optimal activation of antigen-presenting cells responsible for adaptive immunity.

The initial work with AS04 paved the way for a detailed evaluation of the mode of action of AS03 and AS01. These Adjuvant Systems induce local and transient innate responses, but of a different magnitude.^102,103^ Correlated with its ability to induce a higher CMI response, AS01 induced a higher level of transient inflammation as compared to AS03 and AS04. This is due to the synergistic effect between QS-21 and MPL in AS01,^83^ an effect that is not observed between MPL and aluminum in AS04. A common feature among the Adjuvant Systems is their ability to increase the number of activated CD11c+ dendritic cells in the draining lymph node. Ex-vivo experiments demonstrated that those cells were the main driver of antigen-specific T-cell priming, thereby validating the concept that they are key cells in bridging innate and adaptive immunity. The presence of α-tocopherol in AS03 was also required to achieve an enhanced antibody response, and modulated the innate response and the antigen uptake in monocytes, a cell type that is preferentially recruited by AS03.

These data, that were for most part, generated after their initial formulation and clinical development, demonstrate the original concept that combining immunostimulants in adjuvants generates diverse and sometimes synergistic signals required to generate higher immune response to the antigen.

## Lessons and conclusions

The design and development of Adjuvant Systems began 25 y ago and continues to this day. During the investigational process, key lessons were learnt that proved critical for the success of the approach: the most important of these was to start with the right antigen to be combined with the appropriate adjuvant to give rise to the most suitable vaccine. Adjuvant Systems were discovered through serendipity at a time when available knowledge did not allow the establishment of their mode of action. It was through a rational and rigorous scientific approach that their mode of action and safety profile has been assessed. The increased understanding of the mechanism of action of Adjuvant Systems is allowing scientists to venture into new areas, such as the development of therapeutic vaccines targeting cancers or chronic disorders. Above all, an increasingly diverse panel of technologies is being deployed to address the medical needs posed by current and emerging infectious diseases. New knowledge and tools such as epigenetics, vaccinomics and new antigen discovery technologies, have stimulated a rethinking of the adequacy of some currently available vaccines and the development of better vaccines and vaccines effective in specific populations such as infants, the elderly and the immune-compromised. This will pave the way to a true vaccinology approach based on the combination of expertise from numerous disciplines, all of which will be critical to the advancement of vaccine science: none enough on their own.

## Trademarks

Fendrix, Cervarix, Arepanrix, and Pandemrix are trademarks of the GSK group of companies.

## Abbreviations

AS: adjuvant system
CMI: cell-mediated immunity
CpG7909: an immunostimulatory nucleotide
HBV: hepatitis B virus
HIV: human immunodeficiency virus
HPV: human papillomavirus
HSV: Herpes simplex virus
IFN-γ: interferon-gamma
MPL: 3-deacylated monophosphoryl lipid
QS-21: Quillaja saponaria Molina: fraction 21 (Antigenics Inc., a wholly owned subsidiary of Agenus Inc., Lexington, MA, USA)
TLR: toll-like receptor
